# Comparison of glycoside hydrolase family 3 β-xylosidases from basidiomycetes and ascomycetes reveals evolutionarily distinct xylan degradation systems

**DOI:** 10.1016/j.jbc.2022.101670

**Published:** 2022-02-01

**Authors:** Keisuke Kojima, Naoki Sunagawa, Nils Egil Mikkelsen, Henrik Hansson, Saeid Karkehabadi, Masahiro Samejima, Mats Sandgren, Kiyohiko Igarashi

**Affiliations:** 1 Department of Biomaterial Sciences, Graduate School of Agricultural and Life Sciences, The University of Tokyo, Bunkyo-ku, Tokyo, Japan; 2 Department of Molecular Sciences, Swedish University of Agricultural Sciences, Uppsala, Sweden; 3 Faculty of Engineering, Shinshu University, Nagano, Japan; 4 VTT Technical Research Centre of Finland, Espoo, Finland

**Keywords:** glycoside hydrolase family 3, β-xylosidase, xylan, *Phanerochaete chrysosporium*, *Trichoderma reesei*, Bgl, β-glucosidase, Bxl, xylan 1,4-β-xylosidase, CAZy, Carbohydrate-Active enZymes, DP, degree of polymerization, FnIII, fibronectin III, GH, glycoside hydrolases, *Hv*ExoI, β-d-exohydrolase from *Hordeum vulgare*, JGI, Joint Genome Institute, *Km*Bgl3, GH3 Bgl from *Kluyveromyces marxianus*, *Pc*Bxl3, GH3 Bxl from *P. chrysosporium*, PDB, Protein Data Bank, *Tr*Cel3A, GH3 Bgl from *T. reesei*, *Tr*Xyl3A, GH3 Bxl from *T. reesei*, UXX, 2^3^-α-d-glucuronyl-xylotriose, X_2_, xylobiose, X_3_, xylotriose, X_4_, xylotetraose, X_5_, xylopentaose, XA^3^, 1^3^-α-l-arabinofuranosyl-xylobiose, XAc^3^, 1^3^-acetyl xylobiose, XAXX, 3^3^-α-l-arabinofuranosyl-xylotetraose, XA^2^XX, 2^3^-α-l-arabinofuranosyl-xylotetraose, XUXX, 2^3^-α-d-glucuronyl-xylotetraose

## Abstract

Xylan is the most common hemicellulose in plant cell walls, though the structure of xylan polymers differs between plant species. Here, to gain a better understanding of fungal xylan degradation systems, which can enhance enzymatic saccharification of plant cell walls in industrial processes, we conducted a comparative study of two glycoside hydrolase family 3 (GH3) β-xylosidases (Bxls), one from the basidiomycete *Phanerochaete chrysosporium* (*Pc*Bxl3), and the other from the ascomycete *Trichoderma reesei* (*Tr*Xyl3A). A comparison of the crystal structures of the two enzymes, both with saccharide bound at the catalytic center, provided insight into the basis of substrate binding at each subsite. *Pc*Bxl3 has a substrate-binding pocket at subsite -1, while *Tr*Xyl3A has an extra loop that contains additional binding subsites. Furthermore, kinetic experiments revealed that *Pc*Bxl3 degraded xylooligosaccharides faster than *Tr*Xyl3A, while the *K*_M_ values of *Tr*Xyl3A were lower than those of *Pc*Bxl3. The relationship between substrate specificity and degree of polymerization of substrates suggested that *Pc*Bxl3 preferentially degrades xylobiose (X_2_), while *Tr*Xyl3A degrades longer xylooligosaccharides. Moreover, docking simulation supported the existence of extended positive subsites of *Tr*Xyl3A in the extra loop located at the N-terminus of the protein. Finally, phylogenetic analysis suggests that wood-decaying basidiomycetes use Bxls such as *Pc*Bxl3 that act efficiently on xylan structures from woody plants, whereas molds use instead Bxls that efficiently degrade xylan from grass. Our results provide added insights into fungal efficient xylan degradation systems.

Woody and herbaceous biomasses are sustainable sources of biofuel and bio-based chemicals that can replace fossil resources, and enzymatic saccharification is a mild and effective way to utilize them. In nature, fungi degrade polysaccharides such as cellulose and hemicellulose, which account for about 70% of plant cell walls, with producing a variety of enzymes to support their growth. Therefore, the characterization of these enzymes can provide an insight into not only fungal degradation system, but also efficient enzymatic saccharification of the cellulosic biomass. Since some hemicelluloses are present on cellulose crystals, degradation of hemicellulose is crucial. Xylan is a common hemicellulose in the secondary cell wall of Angiosperms (1). The main chain of xylan is composed of β-1,4-linked xylose residues, and the degree of polymerization (DP) is about 80 to 150, regardless of the species and tissue. In contrast, the polysaccharides decorating the main chain differ greatly among plant species. Fungi produce various enzymes to mediate degradation of xylan into xylose (2). These enzymes are classified into two types, of which one degrades the main chain and the other degrades the side chains (3). The xylan main chain is degraded into xylooligosaccharides with low DPs by xylanase, and these products are further degraded into xylose by β-xylosidases (Bxls). In contrast to this simple degradation system, the digestion of polysaccharides decorating the main chain differs depending on their nature. Many kinds of enzymes are involved in cleaving polysaccharides attached to the main chain, including arabinofuranosidases, glucuronidases, and acetyl esterases.

Bxls are key enzymes for fungi to utilize xylan, because, unlike xylanase, they efficiently produce the monosaccharide xylose. Although Bxl works at the end of the xylan degradation system, it has to degrade a variety of substrates because xylanases produce diverse types of small xylooligosaccharides with various DPs and side chains, depending on the type of target polysaccharide (4).

Glycoside hydrolases (GH) family 3 (GH3) is one of the largest families in the Carbohydrate-Active enZymes (CAZy) database http://www.cazy.org/) (5) and includes enzymes such as β-glucosidase (Bgl), N-acetylhexosaminidase, oligoxyloglucan β-glycosidase, and β-xylosidase (Bxl). All of them are exo-acting enzymes, which cleave one residue at a time from the nonreducing end of oligo- or polysaccharides, and their substrate specificity is determined by the architecture at subsite -1. It has also been reported that the DP of the substrate is important for substrate specificity. In kinetic studies with the *Phanerochaete chrysosporium* GH3 enzyme Bgl3A, for example, longer laminarioligosaccharides are better substrates (6). In the case of the GH3 Bgl from *Kluyveromyces marxianus* (*Km*BglI), the PA14 domain supports binding of longer substrates (7). But, in contrast to Bgls, little is known about either the substrate specificities of Bxls toward various xylooligosaccharides or the Bxl subsite structures.

The GH3 Bxl from the ascomycete *Hypocrea jecorina* (anamorph, *Trichoderma reesei*) (8), *Tr*Xyl3A, is one of the best-studied GH3 enzymes as regards substrate specificity, especially its activity toward substituted xylooligosaccharides (9). *T. reesei* has an arsenal of enzymes well suited for the degradation of grass biomass (10). For instance, *T. reesei* has several xylanases that belong to GH families 10, 11, and 30 (11, 12, 13). However, it has only one Bxl, named *Tr*Xyl3A, which belongs to GH3. *Tr*Xyl3A can degrade some substituted xylooligosaccharides, such as 2^3^-α-d-glucuronyl-xylotetraose (XUXX) and 2^3^-α-l-arabinofuranosyl-xylotetraose (XA^2^XX) (14). In contrast to ascomycetes fungi, there is limited knowledge about xylan degradation by basidiomycetes. *P. chrysosporium* is one of the best-studied white-rot fungi (15). It produces several xylanases belonging to GH families 10 (GH10) and 11 (GH11) (16), as well as a GH3 Bxl, *Pc*Bxl3, and degrades hardwood. The xylan-degrading enzymes of *P. chrysosporium* have not yet been well characterized.

Here, we describe structural and kinetic comparisons of two β-xylosidases from *P. chrysosporium* and *T. reesei*. The ligand-bound structures of these two enzymes account well for the kinetic differences, which are consistent with the evolution of the different xylan-degrading machineries required to degrade the preferred growth substrates of basidiomycetes and ascomycetes.

## Results

### Sequence analysis

**Figure 1:**
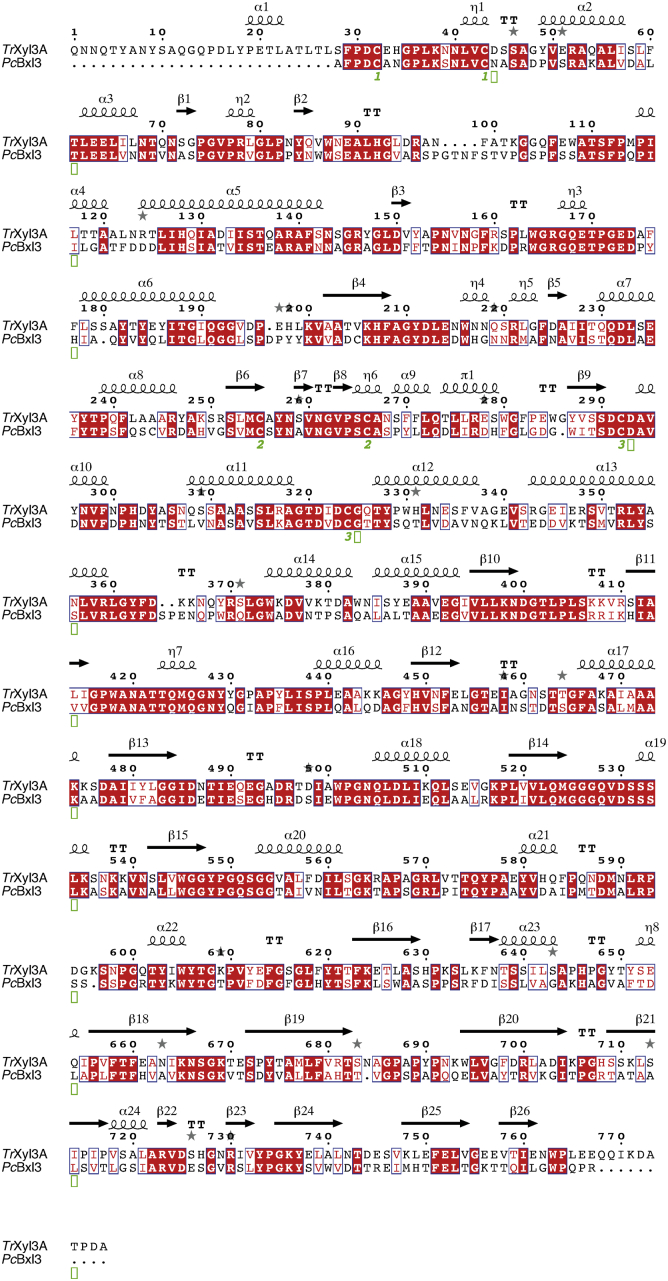
**Comparison of predicted secondary structures.** The amino acid sequences of *Pc*Bxl3 and *Tr*Xyl3A were aligned by Clustal W (42), as shown in Fig. S1. Prediction of secondary structures was conducted by ESPript using the alignment and the native form of *Tr*Xyl3A (43).

*Pc*Bxl3 and *Tr*Xyl3A contain 743 and 777 amino acid residues, respectively. Since the sequence identity between the two enzymes is 54%, the distributions of secondary structures in the two GH3 enzymes are quite similar, as shown in Figure 1. The most significant difference between the two GH3 β-xylosidase structures is an α-helix located at the N-terminal region of *Tr*Xyl3A. This additional N-terminal region containing the α-helix of *Tr*Xyl3A consists of 27 amino acids. There are also ten extra amino acids at the C-terminal region of *Tr*Xyl3A, but no secondary structure is predicted for them.

### Overall structures of *Pc*Bxl3 and *Tr*Xyl3A

To uncover the structural differences between *Pc*Bxl3 and *Tr*Xyl3A, the crystal structures of both enzymes were solved. The crystal structure of *Tr*Xyl3A bound with thioxylobiose was determined by molecular replacement (MR) using free *Tr*Xyl3A (Protein Data Bank (PDB) ID: 5A7M, chain B) as a search model, and the crystal structure of free *Pc*Bxl3 was determined by MR using *Tr*Xyl3A (5AE6 chain B). The structure of *Pc*Bxl3 with xylose bound in the catalytic center was also determined. The data collection statistics and structural refinement statistics are summarized in Table 1.

**Table 1:** Data collection and refinement summary

Data	*Pc*Bxl3	*Pc*Bxl3 bound with X	*Tr*Xyl3A bound with thioxylobiose
Wavelength	1.000	1.000	1.038
Resolution range	44.44–2.54 (2.631–2.54)	44.08–3.08 (3.19–3.08)	28.89–2.1 (2.175–2.1)
Space group	P 21 21 21	P 21 21 21	P 21 21 2
Unit cell (a/b/c)	79.667/91.292/107.082	79.367/91.071/106.012	100.244/202.443/82.441
Unit cell (α/β/γ)	90/90/90	90/90/90	90/90/90
Total reflections	52,707 (5163)	29,463 (2896)	192,986 (19,158)
Unique reflections	26,406 (2587)	14,756 (1450)	98,434 (9738)
Multiplicity	2.0 (2.0)	2.0 (2.0)	2.0 (2.0)
Completeness (%)	99.94 (99.92)	99.91 (99.93)	99.79 (99.85)
*R*_merge_	0.06141 (0.3395)	0.09164 (0.3472)	0.04487 (0.1965)
Reflections used in refinement	26,401 (2586)	14,750 (1449)	98,400 (9738)
Reflections used for *R*_free_	1262 (127)	720 (71)	4910 (478)
*R*_work_	0.1835 (0.2554)	0.2005 (0.2501)	0.1761 (0.2088)
*R*_free_	0.2369 (0.3252)	0.2562 (0.3378)	0.2123 (0.2480)
Number of nonhydrogen atoms	6140	6161	13,404
Macromolecules	5623	5609	11,971
Ligands	118	339	482
Solvent	399	213	951
RMS (bonds)	0.004	0.003	0.007
RMS (angles)	1.06	0.73	1.15
Ramachandran favored (%)	95.55	93.79	96.65
Ramachandran allowed (%)	3.51	5.4	3.16
Ramachandran outliers (%)	0.94	0.81	0.2
Rotamer outliers (%)	0	0	0
Average B-factor	34.54	42.34	22.87
Macromolecules	34.16	41.24	22
Ligands	52.37	64.63	38
Solvent	34.7	35.94	26.23
PDB code	7VC6	7VC7	5AE6

Statistics for the highest-resolution shell are shown in parentheses.

This table was created using the Phenix program.

**Figure 2:**
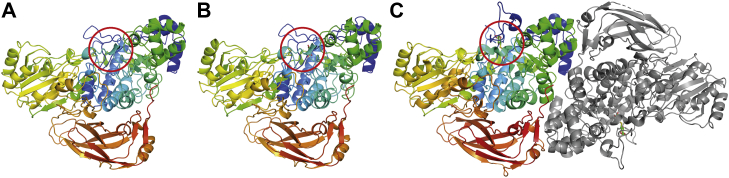
**Overall structures of *Pc*Bxl3 and *Tr*Xyl3A.** Illustration of overall structures of free *Pc*Bxl3 (*A*) and *Pc*Bxl3 bound with xylose (*B*). The latter was obtained from the crystal soaked in 40 v/v % PEG 400 and 100 mM X_2_ for a short time. *Tr*Xyl3A bound with thioxylobiose (*C*) is a protein dimer. Chain A is *gray*. Chain B is *multicolored*. *Red circle* shows the active center. Refer to Figure 3 for the active center in detail.

*Pc*Bxl3 consists of three domains: (β/α)_8_ domain, (α/β)_6_-sandwich domain, and fibronectin III (FnIII)-like domain. The overall structure and order of these domains (illustrated in Fig. 2) are the same as those of *Tr*Xyl3A. The FnIII-like domain is connected to the active domain by a linker, as in other GH3 enzymes. There is a single molecule of *Pc*Bxl3 in the asymmetric unit, whereas there is a protein dimer in the case of *Tr*Xyl3A. *N*-Acetylglucosamine moieties were found at Asn17, Asn40, Asn74, Asn279, Asn286, Asn398, Asn432, and Asn437 of free *Pc*Bxl3.

**Figure 3:**
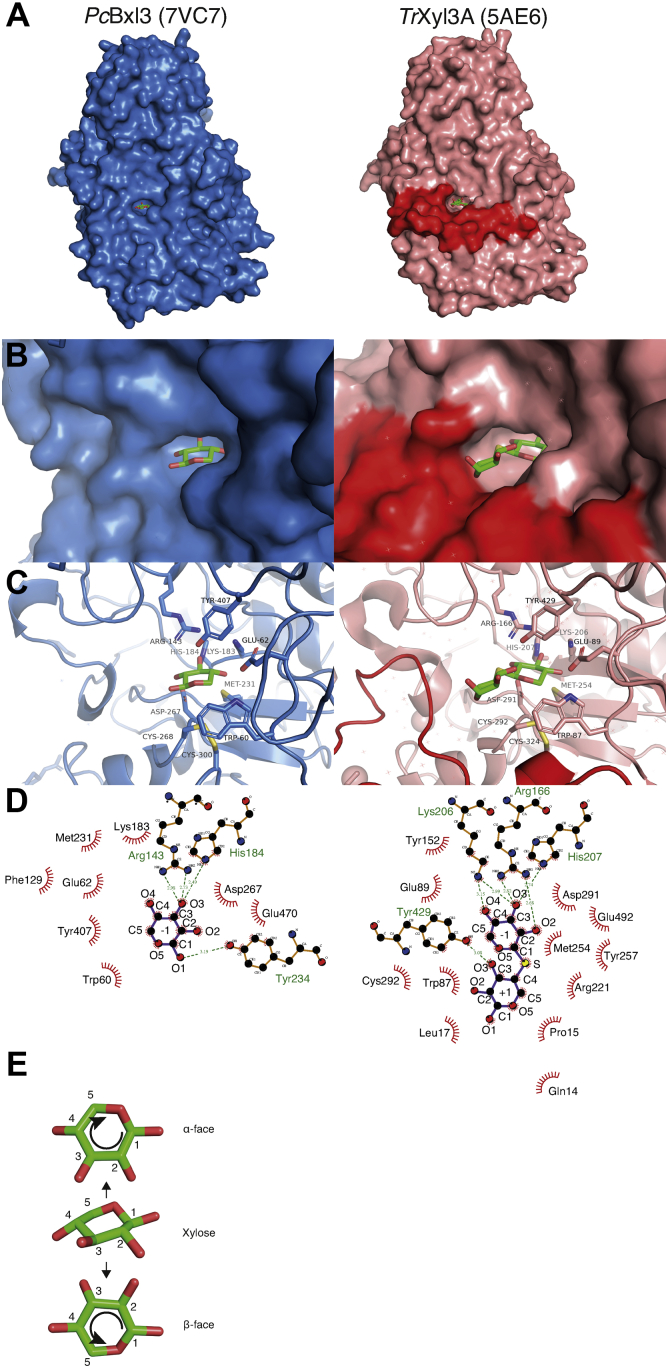
**The structure of the active center.***A*, the surface of *Pc*Bxl3 and *Tr*Xyl3A. *Pc*Bxl3 and *Tr*Xyl3A are colored *sky blue* and *salmon*, respectively. Xylose and thioxylobiose are represented as *green* (carbon), *red* (oxygen), and *yellow* (sulfur) *sticks*. The *red circle* shows the extra loop of *Tr*Xyl3A. *B*, the hole-shaped active center. Representative small compounds are the same as in *A*. The surfaces of *Pc*Bxl3 and *Tr*Xyl3A are shown in *sky blue* and *salmon*, respectively. The *red region* is the extra loop of *Tr*Xyl3A. *C*, the hydrophilic residues within 3.0 Å from substrates and hydrophobic residues around substrates are shown as *sticks*. Oxygen, nitrogen, and sulfur are colored *red*, *blue*, and *yellow*, respectively. Carbon is colored *green*. *D*, the ligand–protein diagrams were created using LigPlot+ (63). *E*, the definition of α- and β-faces of the xylose ring is based on the proposed rule (20).

Focusing on the N-terminal region, an extra loop containing the α-helix is found near the active center of *Tr*Xyl3A, as shown in Figure 3*A*, which is consistent with the results of the secondary structure prediction. In contrast, both enzymes were found to have the C-terminal at the same location even though the secondary structure comparison predicted that *Tr*Xyl3A would have a longer C-terminal region.

### Comparison of the active center

The catalytic residues of GH3 enzymes are glutamic acid and aspartate (17, 18, 19). Superposition of our enzymes on β-d-exohydrolase from *Hordeum vulgare* (*Hv*ExoI, 1IEX) suggests that *Pc*Bxl3 has Glu470 as a possible acid/base amino acid and Asp267 as a catalytic nucleophile, while the corresponding residues in *Tr*Xyl3A are Glu492 and Asp291. The conformation of Glu470 (*Pc*Bxl3) is the same in the native and liganded structures of *Pc*Bxl3, but Glu492 (*Tr*Xyl3A) has different conformations in native and liganded *Tr*Xyl3A.

As shown in Figure 3*B*, both Bxls have a pocket at subsite -1. The binding mode of a xylose residue at subsite -1 is important for substrate recognition and to maintain the substrate in a suitable conformation during the catalytic reaction. There are three important interactions, as illustrated in Figure 3, *C* and *D*: the interactions with the C2, C3, and C4 hydroxyl groups (O2, O3, and O4, respectively), the interaction with C5, and the interactions with the α- and β-faces of the xylose ring, which are named according to the usual rules (20), as shown Figure 3*E*. The hydroxyl groups interact with hydrophilic amino acids. For instance, Asp267 in *Pc*Bxl3 is located at a distance of 2.7 Å from O2 of the xylose moiety bound at subsite −1, while Arg166 and Asp291 in *Tr*Xyl3A are located at distances of 2.7 Å and 2.8 Å, respectively. In addition, Arg143 and His184 are located at distances of 3.0 and 2.4 Å from O3 of the xylose residue at subsite −1, respectively. The corresponding amino acids are well conserved in *Tr*Xyl3A: Arg166, Lys206, and His207 are 2.8, 3.0, and 2.7 Å from O3 of the xylose residue, respectively. Furthermore, the amino acids near the C4 hydroxyl group (O4) of the xylose residue bound at subsite −1 are also similar in the two enzymes. Glu62 and Lys183 are located at distances of 2.7 and 3.0 Å from O4 of the xylose residue in *Pc*Bxl3, respectively, and Glu89 and Lys206 in *Tr*Xyl3 are both located at a distance of 2.7 Å from O4. In addition, Trp60 of *Pc*Bxl3 and Trp87 of *Tr*Xyl3A are located near C5. Thus, tryptophan interacts with C5 hydrophobically. The environments opposite the α- and β-faces of the xylose ring are significantly different. The hydroxyl group of Tyr407 of *Pc*Bxl3 and that of Tyr429 of *Tr*Xyl3A are oriented toward the α-face, while a disulfide bond and methionine are exposed to the β-face. The amino acids surrounding the nonreducing end sugar moiety are well conserved, but *Pc*Bxl3 has Phe129 as the residue corresponding to Tyr152 of *Tr*Xyl3A, indicating that subsite −1 of *Pc*Bxl3 would be more hydrophobic than that of *Tr*Xyl3A.

In *Pc*Bxl3, there is no clearly detectable subsite +1 (Fig. 3). In contrast, *Tr*Xyl3A covers a half of the xylose residue bound at subsite +1. This difference is attributable to the abovementioned extra loop of *Tr*Xyl3A located adjacent to the active site of *Tr*Xyl3A. At the side of O3 of the xylose residue at subsite +1, there is a small space. This is consistent with the fact that *Tr*Xyl3A can degrade 1^3^-α-l-arabinofuranosyl-xylobiose (XAc^3^), but not 1^3^-acetyl xylobiose (XA^3^) (21). Compared with GH3 Bgls such as *Hv*ExoI (1IEX) (17) and Bgl from *T. reesei* (*Tr*Cel3A, 4I8D) (22), subsites of Bxls do not fully cover the disaccharides.

### Comparison of kinetic parameters

Kinetic analysis was conducted to examine the substrate specificities toward xylobiose (X_2_), xylotriose (X_3_), xylotetraose (X_4_), xylopentaose (X_5_). DPs of them are thought to be those of major products of xylan degradation by xylanases. As expected from the structural differences between *Pc*Bxl3 and *Tr*Xyl3A, the kinetic parameters are significant different (Table 2), *i.e.*, the values of *k*_cat_ toward X_2_, X_3_, X_4_, and X_5_ are in range from 1.66 to 2.05 s^−1^ for *Pc*Bxl3, while those of *Tr*Xyl3A range from 0.34 to 0.82 s^−1^, about a quarter of those of *Pc*Bxl3. The *k*_cat_ of *Tr*Xyl3A is DP-dependent, increasing up to DP 4 and then decreasing at DP 5. In contrast, the *K*_M_ values of *Pc*Bxl3 are higher than those of *Tr*Xyl3A. The measured *K*_M_ values of *Pc*Bxl3 range from 0.74 to 1.21 mM, while those of *Tr*Xyl3A range from 0.024 to 0.09 mM, a tenth of those of *Pc*Bxl3. Thus, these two enzymes have very different catalytic characteristics.

**Table 2:** Kinetic parameters of *Pc*Bxl3 and *Tr*Xyl3A for xylooligosaccharides with various DPs

Parameters	*Pc*Bxl3			*Tr*Xyl3A		
	*k*_cat_ (s^−1^)	*K*_M_ (mM)	*k*_cat_/*K*_M_ (mM^−1^s^−1^)	*k*_cat_ (s^−1^)	*K*_M_ (μM)	*k*_cat_/*K*_M_ (mM^−1^s^−1^)
X_2_	1.81 ± 0.05	0.90 ± 0.06	2.0 ± 0.2	0.34 ± 0.01	90 ± 10	3.7 ± 0.6
X_3_	1.80 ± 0.06	1.14 ± 0.08	1.6 ± 0.2	0.39 ± 0.01	24 ± 5	16 ± 4
X_4_	2.05 ± 0.05	0.74 ± 0.04	2.8 ± 0.2	0.82 ± 0.03	70 ± 10	12 ± 2
X_5_	1.66 ± 0.15	1.21 ± 0.25	1.4 ± 0.3	0.56 ± 0.01	66 ± 3	8.5 ± 0.5

The final concentrations of *Pc*Bxl3 and *Tr*Xyl3A were 30.2 nM and 26.6 nM, respectively. The reaction solution contained 50 mM sodium acetate buffer (pH 5.0). The reaction was performed at 30 °C for 30 min and stopped by heating the mixture at 95 °C for 5 min. The amount of released xylose was determined using HPLC with a Corona CAD detector.

**Figure 4:**
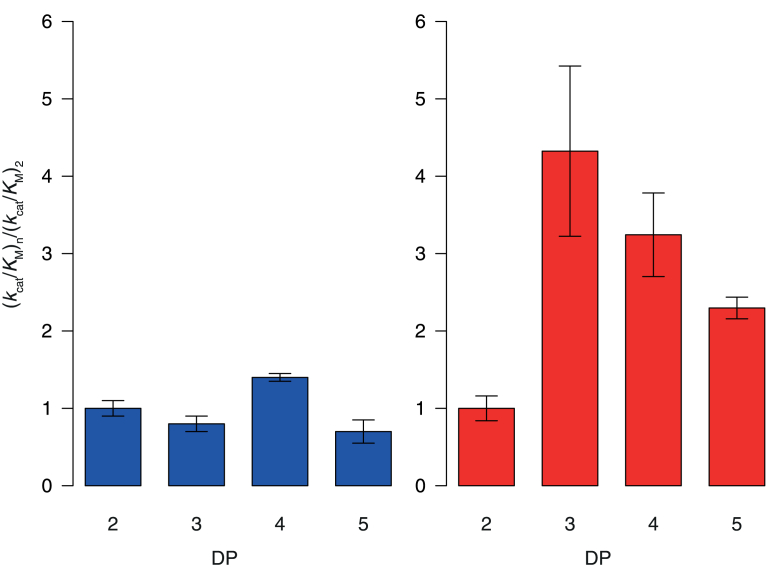
R**elationships between substrate specificity and substrate DP value.** The ratio of the value of (*k*_cat_/*K*_M_)_n_ to that of (*k*_cat_/*K*_M_)_2_ was calculated (see Table 1). *Blue bars*, *Pc*Bxl3; *red bars*, *Tr*Xyl3A.

The calculated *k*_cat_/*K*_M_ values, representing catalytic efficiency, indicate that *Tr*Xyl3A is two to ten times more efficient than *Pc*Bxl3, though direct comparison is difficult since the enzymes have different reaction characteristics. Thus, for further evaluation of their substrate specificity, the DP dependence of *k*_cat_/*K*_M_ was investigated (Fig. 4). The DP of xylooligosaccharides had little effect on the *k*_cat_/*K*_M_ value of *Pc*Bxl3 compared with that of *Tr*Xyl3A. The *k*_cat_/*K*_M_ of *Tr*Xyl3A, in contrast, increased with increasing DP of the substrate. Longer xylooligosaccharides than X_2_ were better substrates for *Tr*Xyl3A and X_3_ was the best substrate. Thus, the kinetics parameters suggest that *Pc*Bxl3 degrades different xylooligosaccharides equally well, whereas *Tr*Xyl3A preferentially degrades longer oligosaccharides.

### Subsite affinities

**Figure 5:**
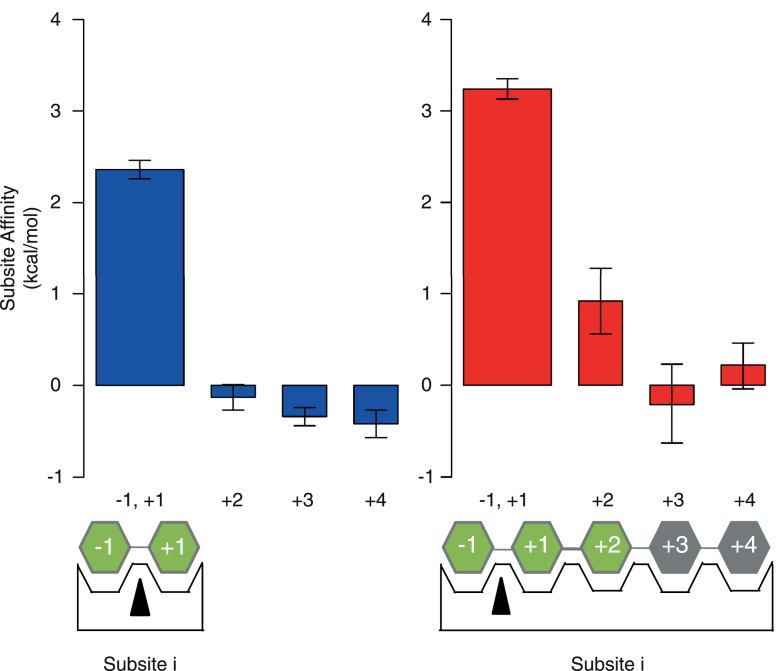
**Summary of subsite affinities.** Subsite affinities were calculated from *k*_cat_/*K*_M_ in Table 1 by Hiromi’s method (23, 24, 25). *Blue bars*, *Pc*Bxl3; *red bars*, *Tr*Xyl3A.

The subsite affinities (23) of both Bxls were calculated from the kinetic parameters. In the case of exo-type enzymes such as Bxl, the ratio of (*k*_cat_/*K*_M_)_n+1_/(*k*_cat_/*K*_M_)_n_ reflects the binding affinity at subsite n. Based on the maximum value of *k*_cat_, *k*_int_ of *Pc*Bxl3 and *Tr*Xyl3A was assumed to be 2.05 and 0.82 s^−1^, respectively. The results are shown in Figure 5. We attempted to calculate the affinities of subsites −1 and +1 according to the methods of Hiromi and coworkers (24, 25), but without success. To obtain the subsite affinity at subsite −1, it is necessary to assume that there are two binding modes, productive and nonproductive (25). However, this would not be the case for *Pc*Bxl3. On the assumption of two binding modes, the subsite affinity at subsite −1 can be derived from *k*_cat_ and the affinities of subsites +2 to +4 (25). However, this does not work in the present case, because there are very weak affinities at subsite +3 and +4 in *Pc*Bxl3. Therefore, since our focus here is on the relationship between DP and Bxl function for DP = 2 or more, we used the sum of the affinities of subsites −1 and +1 as a parameter for comparison. As expected from the results for other glycosidases, the active center (sum of subsites −1 and +1) has a higher affinity than the sum of the affinities of other subsites. *Tr*Xyl3A shows higher affinity at the active center than *Pc*Bxl3, and the difference is about 1 kcal/mol, which is consistent with the structural findings.

Interestingly, subsite +2 of *Tr*Xyl3A has a binding energy of 1 kcal/mol, which is typical for subsites of other GH (6, 25, 26, 27, 28, 29), whereas the binding energy at this subsite of *Pc*Bxl3 was very low, and subsite +3 and +4 also showed little affinity. These results indicate that *Pc*Bxl3 has at most two substrate-binding subsites, *i.e.*, subsite −1 and subsite +1, whereas *Tr*Xyl3A has three, *i.e.*, subsites -1, +1, and +2.

### Docking simulation

**Figure 6:**
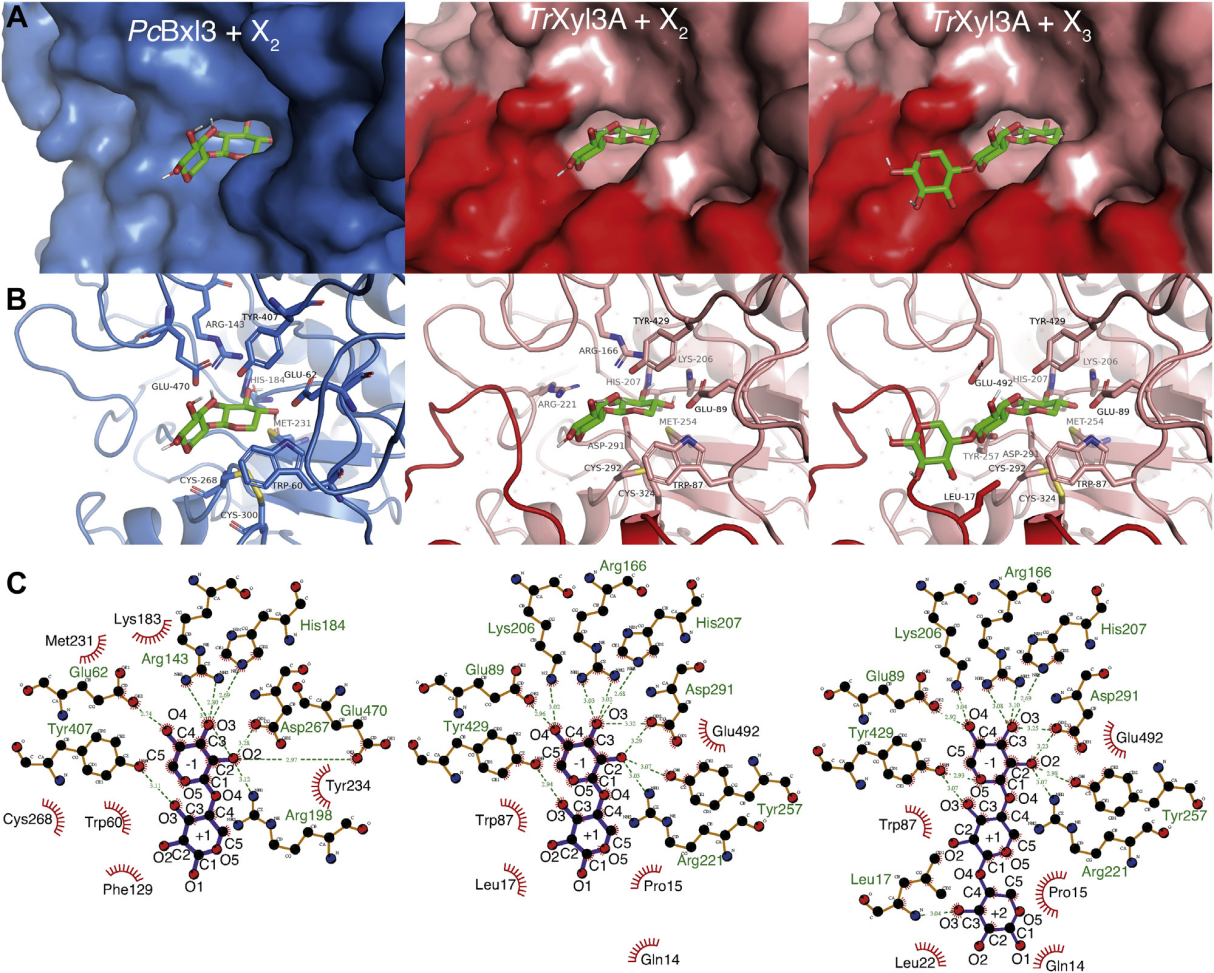
**Docking simulation.** The model structures of X_2_ and X_3_ were created using the SWEET2 server (66, 67). Docking simulation was done by Autodock Vina (64). Substrates are represented as *green* (carbon) and *red* (oxygen) *sticks*. *A*, the surface and sticks of *Pc*Bxl3 and *Tr*Xyl3A are shown in *sky blue* and *salmon*, respectively. The surface of the extra loop of *Tr*Xyl3A is *red*. The *sticks* and the extra loop are shown in *red*. *B*, hydrophilic residues near the substrate (within 3.0 Å) and hydrophobic residues around the substrates are shown as *sticks*. The colors are the same as in Figure 3*B*. Water molecules are shown as *red asterisks*. *C*, ligand–protein diagrams were created using LigPlot+ (63).

Next, the affinities of xylose and X_2_ (*Pc*Bxl3 and *Tr*Xyl3A), and X_3_ (*Tr*Xyl3A) were estimated by docking simulation; the results are summarized in Table 3 and visualized in Figure 6. While some simulations showed different modes of binding, most of the highest scores were recorded for the same binding mode as that of the xylose residue in the crystal structure of *Pc*Bxl3 and as that of thioxylobiose in the crystal structure of *Tr*Xyl3A, supporting the reliability of the simulation results. In the case of *Pc*Bxl3, X_2_ showed the highest score, −6.3 kcal/mol. In the case of *Tr*Xyl3A, X_2_ and X_3_ showed the highest score, −7.9 and −8.9 kcal/mol, respectively. The difference between the scores of the X_2_ and X_3_ binding modes is -1.0 kcal/mol, which is consistent with the subsite affinity in Figure 5, while the difference between the scores of the Xyl and X_2_ binding modes is -1.7 kcal/mol.

**Table 3:** Scores from docking simulation

*Pc*Bxl3	Affinity (kcal/mol)		*Tr*Xyl3A	Affinity (kcal/mol)		
Binding mode	Xylose	X_2_	Binding mode	Xylose	X_2_	X_3_
1	−5.8	−6.3	1	−6.2	−7.9	−8.9
2	−5.6	−6.3	2	−6.1	−7.5	−8.6
3	−5.3	−6.1	3	−5.4	−7.3	−8.2
4	−4.9	−5.9	4	−5.2	−7.1	−8.1
5	−4.6	−5.7	5	−5.1	−6.9	−7.9
6	−4.1	−5.1	6	−5.1	−6.9	−7.8
7	−4.0	−5.1	7	−4.9	−6.8	−7.0
8	−3.9	−5.0	8	−4.9	−6.7	−7.0

Comparing the scores of the xylose and X_2_ binding modes of *Pc*Bxl3 and *Tr*Xyl3A, the score of *Pc*Bxl3 is about a half of that of *Tr*Xyl3A. As shown in Figure 6*B*, only O3 of the xylose residue at subsite +1 can have a hydrophilic interaction with Tyr407 (*Pc*Bxl3) and Tyr429 (*Tr*Xyl3A). Further, as shown in Figure 6*C*, less than four atoms of the xylose residue at subsite +1 interact with *Pc*Bxl3, whereas six atoms of xylose with the residue at subsite +1 interact with *Tr*Xyl3A. Thus, the difference between the scores is thought to be mainly due to the difference in number of hydrophobic interactions at subsite +1.

Focusing on *Tr*Xyl3A docked with X_3_ in Figure 6*B*, Leu17 is located at a distance of 3.0 Å from C5 of the xylose moiety at subsite +2. Additionally, two water molecules on the surface of the extra loop are placed near the xylose moiety, at a distance of 2.9 Å. They may have a role in binding the substrate, because the results of docking simulation without water gave a significantly lower score (data not shown). These results support that the idea *Tr*Xyl3A contains subsite +2 and suggest that the extra loop is important for binding longer oligosaccharides.

## Discussion

### Comparison of GH3 enzymes

**Figure 7:**
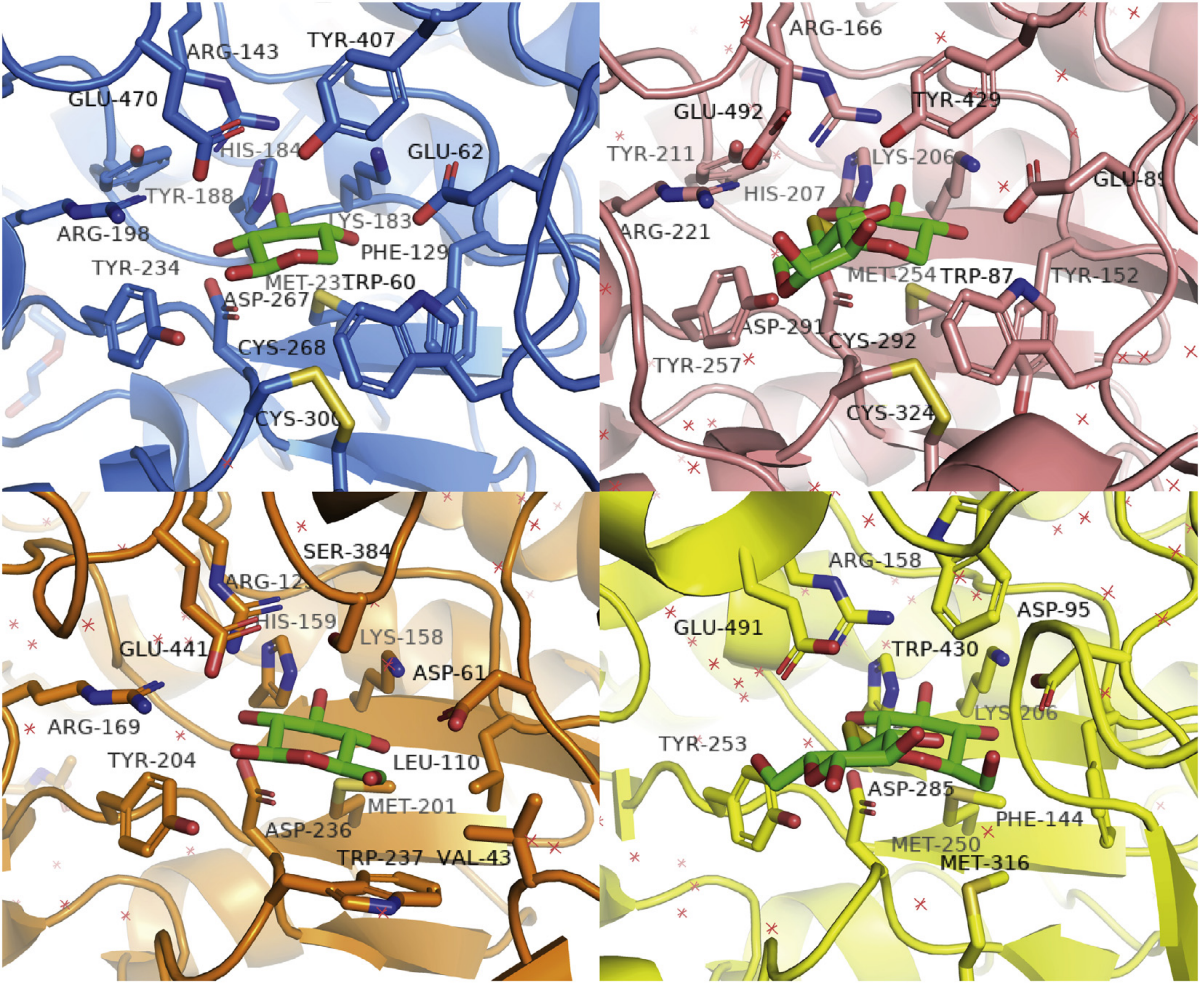
**Comparison of the active centers of GH3 Bxls and Bgls.** The active centers of *Pc*Bxl3 (PDB ID: 7VC7, *sky blue*), *Tr*Xyl3A (5AE6, *salmon*), *Tr*Cel3A bound with glucose (4I8D, *orange*) (22) and *Hv*Exo1 bound with thiocellobiose (1IEX, *yellow*) (17) are shown as *cartoons*. The amino acids around the active centers are shown as *sticks*, and the *colors* are the same as in Figures 3*B* and 6*B*.

**Figure 8:**
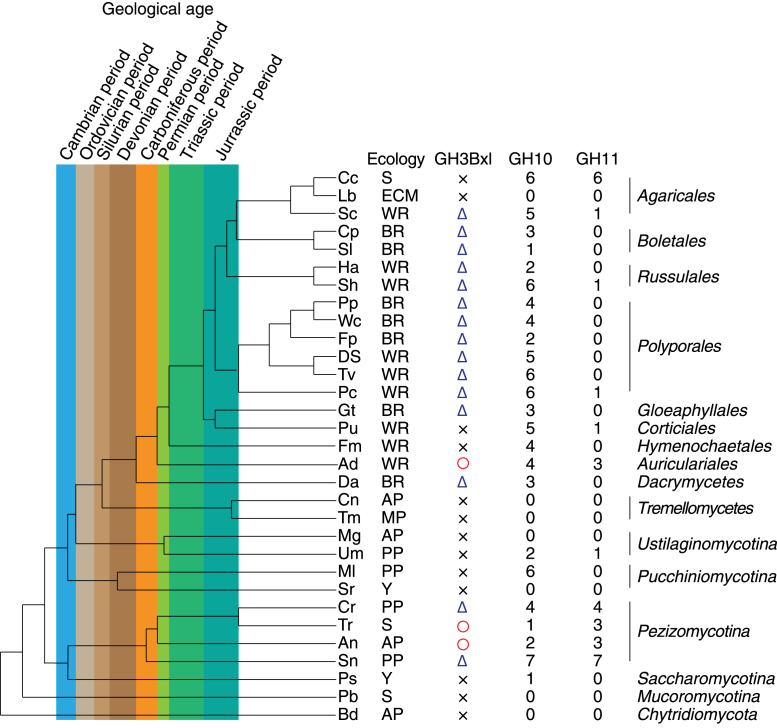
**Phylogenetic tree and xylan degradation enzymes.** The phylogenetic tree and the numbers of GH 10 and 11 enzymes are based on previous research (33). *Cross*, *triangle*, and *circle symbols* indicate no GH 3Bgl, possession of Bxl lacking the extra loop, and possession of Bxl with the extra loop, respectively. S, ECM, WR, BR, AP, MP, PP, and Y in the Ecology column mean non-wood-decaying saprotroph, mycorrhiza, white-rot, brown-rot, animal pathogen/parasite, mycoparasite, plant pathogen, and yeast, respectively. Refer to the previous paper (33) for fungal names.

The substrate specificity of GH3 enzymes is mainly determined by subsite −1, because exo-type enzymes must recognize and bind the residue at the nonreducing end throughout the reaction. Thus, subsite −1 of enzymes that have similar substrate specificities generally consists of the same or similar amino acid residues. Figure 7 shows the structure of the active center of four GH3 enzymes: *Pc*Bxl3 (7VC7), *Tr*Xyl3A (5AE6), *Tr*Cel3A (4I8D) (22) and *Hv*ExoI (1IEX) (17). Most of the hydrophilic amino acids interacting with O2, O3, and O4 are common among them. Whether they bind xylose or glucose, the modes of binding are conserved in the GH3 enzymes. A key difference between xylose and glucose is that xylose lacks C6. As shown in Figure 3, Bxl has a tryptophan residue near C5. Since Bgls do not have this tryptophan residue, there is sufficient space for C6. Thus, the tryptophan residue appears to have a role not only in the hydrophobic interaction with C5, but also in substrate selectivity. Furthermore, the environment at the α- and β-faces is different between Bxls and Bgls. As shown in Figure 3, both Bxls have a tyrosine residue on the side of the α-face and disulfide and methionine on the other side. Thus, there is a hydrophobicity gradient. As shown in Figure 8, *Tr*Cel3A has Ser384 on the side of α-face and Try237 and methionine on the other side, while *Hv*ExoI has Trp158 on the side of the α-face and two methionines on the side of the β-face. Bgls also interact with the sugar ring hydrophobically, but only methionine on the β-face is common, and there does not seem to be the same hydrophobicity gradient. These differences provide insight into the binding mode of xylose at subsite -1 of Bxls. Since xylose is more symmetric than glucose, differentiation of the two sides of the ring is more difficult. However, the β-face is more hydrophobic than the α-face because of the difference of axial and equatorial hydrogen at C5. To recognize this difference, there is a gradient of hydrophobicity on both sides in GH3 Bxls. Based on these considerations, the three tyrosine residues in subsite -1 of *Tr*Xyl3A appear to have different roles. Tyr257 is conserved in GH3 Bgls and Bxls. In *Tr*Cel3A, the equivalent tyrosine is thought to have a hydrophilic interaction with O2. As shown in Figure 6, Tyr257 can be located sufficiently near to O2 for hydrogen bonding. Thus, this tyrosine may bind O2. Tyr429 of *Tr*Xyl3A is thought to interact not only with the α-face of the xylose ring as shown in Figure 7, but also with O3 of the xylose residue at subsite +1, depending on the orientation of the xylose residue, as shown in Figure 6. Since Tyr152 of *Tr*Xyl3A is located at a position corresponding to Phe129 of *Pc*Bxl3 (Fig. 3), this tyrosine contributes to the hydrophobic environment.

In contrast to subsite −1 in Bxls, the structures of subsite +1 in *Pc*Bxl3 and *Tr*Xyl3A are significantly different. In the crystal structure of *Pc*Bxl3, subsite +1 was not clearly identified, but that of *Tr*Xyl3A was clearly visualized in the crystal structure. The isoprimeverose (α-d-xylopyranose-(1→6)-d-glucopyranose derived from xyloglucan)-producing enzyme belonging to GH3 from *Aspergillus oryzae* has a shallow pocket adjacent to subsite −1, broadening it to subsite −1’ (30). The pockets of Lin1860 from *Listeria innocua* and BT_3567 protein from *Bacteroides thetaiotaomicron*, which degrade β-1,2-glucooligosaccharides, are also shallow (31, 32). These subsite structures are consistent with a wider range of substrate structures. In the case of Bgls degrading cellooligosaccharides, the glucose moiety at subsite +1 is sandwiched between two tryptophan residues; this is possible because the substrates have a straight chain without substitution. GH3 Bgls have a pocket large enough to bind disaccharides, while GH3 Bxls have a smaller pocket, and *Tr*Xyl3A has the extra loop that contributes to binding longer, substituted xylooligosaccharides.

Furthermore, it was suggested that *Tr*Xyl3A contains subsite +2 because the calculated affinity, +1 kcal/mol, is consistent with that of extra subsites in other enzymes (6, 25, 26, 27, 28, 29). The shape of subsites is also important. For example, in *Km*Bgl3, the subsite specificity is restricted by the PA14 loop (7). In contrast, the extra loop of *Tr*Xyl3A is open. The space around O2 at subsite +1 of *Tr*Xyl3A can accept substitutions such as GlcA and arabinose. However, O3 is more restricted, and it was reported that *Tr*Xyl3A cannot degrade XA^3^XX (14), though an acetyl group at O3 is small enough to permit binding (21). Thus, wood-decaying fungi and soft-plant-decaying fungi have specific Bxls to optimize their xylan-degradation systems.

### Different roles of tyrosine residues around the active center

Based on the above results and discussion, the three tyrosine residues in subsite -1 of *Tr*Xyl3A have different roles. Tyr257 is conserved in GH3 Bgls and Bxls. In *Tr*Cel3A, the corresponding tyrosine is thought to have a hydrophilic interaction with O2. As shown in Figure 6, Tyr257 is located sufficiently near O2 to form a hydrogen bond. Thus, this tyrosine also interacts with O2. Tyr429 of *Tr*Xyl3A is thought to interact not only with the α-face of the xylose ring as shown in Figure 7, but also with O3 of the xylose residue at subsite +1, depending on the orientation of the xylose residue (Fig. 6). Since Phe129 of *Pc*Bxl3 (Fig. 3) is located at a position corresponding to that of Tyr152 of *Tr*Xyl3A, this tyrosine contributes to the hydrophobic environment.

### Relationship between kinetics and structure

Subsite theory can account for the differences in the kinetic parameters of the enzymes studied here. Kinetic analysis revealed that the *k*_cat_ values of *Pc*Bxl3 are generally higher than those of *Tr*Xyl3A. This is probably because *Tr*Xyl3A has more subsites, which would increase the ratio of nonproductive binding. In contrast, the *K*_M_ values of *Tr*Xyl3A are one-tenth of those of *Pc*Bxl3, which translates into higher substrate affinity in general.

In summary, *Pc*Bxl3 degrades xylooligosaccharides with various DPs and prefers X_2_, based on the crystal structure. In contrast, *Tr*Xyl3A degrades longer xylooligosaccharides with specific substitutions (3, 14, 21).

### Xylan degradation strategies

Xylanases produce various substituted xylooligosaccharides (3). Differences in fungal species, the characteristics of their enzymes, and the target xylan structures need to be considered to understand the overall xylan degradation system. Figure 8 shows the phylogenetic tree of 31 fungi based on the study by Floudas *et al.* (33), combined with information on fungal ecology and the numbers of GH10 and GH11 xylanases (33), as well as the presence/absence of GH3 Bxls and the presence/absence of the extra loop. Not all basidiomycetes have GH3 Bxls and among those that do, the enzyme seems to lack the extra loop. There appears to be no major difference between white-rot fungi (WR) and brown-rot fungi (BR). In contrast, Bxls from the ascomycetes *T. reesei* and *Aspergillus niger* (34) have the extra loop region.

Focusing on geological age, it seems likely that fungi and molds acquired GH3 Bxls and GH11 xylanases in the Carboniferous period. These Bxls may have lacked the loop region, such as *Pc*Bxl3, because pteridophytes, which flourished during the Carboniferous period, have xylan in their cell walls (35). Therefore, these results may imply that fungi acquired their xylan-degradation systems in order to utilize pteridophytes as a carbon source. Some fungi, including *Auricularia delicata* and *A. niger*, appear to have acquired GH3 Bxl with the extra loop, such as *Tr*Xyl3A, between the Carboniferous and Permian periods. Subsequently, most fungi adopted Bxls without the loop again, but *T. reesei* retained Bxl with the loop. *Cryphonectria parasitica* is an ascomycete, but has a Bxl without the loop, possibly because it is parasitic on bark. It seems likely that fungi adapted their xylan-degradation systems in response the subsequent evolution of their target plants.

White-rot fungi have GH10 xylanases and favor hardwood substrates. Since the substitution pattern of glucuronic acid is distributed (4) and approximately one in two xylose resides is acetylated (36), acetylglucuronoxylan is resistant to degradation by xylanases (37), and acetyl xylan esterases play an important role (38). If GH10 xylanases and acetyl xylan esterases attack xylan, the final products would be xylose, X_2_ and 2^3^-α-d-glucuronyl-xylotriose (UXX). Thus, any Bxl in this environment should preferentially degrade small xylooligosaccharides. Brown-rot fungi also have GH10 xylanases, but favor softwood substrates, which include arabinoglucuronoxylan. The structure of arabinoglucuronoxylan is simpler than that of acetyl glucuronoxylan in that glucuronic acid substitution mostly occurs once every six xylose residues, and arabinose is located two residues away from a GlcA-substituted residue (39). Thus, GH10 digestion affords xylobiose and xylooligosaccharides substituted with GlcA or Ara at the nonreducing end (39). Bxl in this environment is mainly required to degrade these relatively small oligosaccharides after debranching. In contrast to wood-decaying fungi, soft-rot fungi and molds mainly use GH11 and GH30 xylanases. They degrade wood surfaces and herbaceous biomass. GH11 and GH30 xylanases produce longer xylooligosaccharides than GH10 xylanases. In the case of GH11 xylanases, Bxl needs to degrade X_4_ derived from XUXX and 1^3^-α-l-arabinofuranosyl-xylobiose (XAXX). GH30 xylanases produce longer xylooligosaccharides depending on the substitution patterns. Furthermore, some substituted xylooligosaccharides need to be digested by Bxl before debranching (3). These facts are consistent with the structure of *Tr*Xyl3A, which favors longer xylooligosaccharides.

In this paper, two types of fungal Bxls were characterized and compared with each other. Kinetic analysis taking account of the subsite structure and substrate DP could account for the different substrate specificities of *Pc*Bxl3 and *Tr*Xyl3A. Our results highlight the different fungal tactics employed to degrade xylan: wood-decaying basidiomycetes use Bxls, such as *Pc*Bxl3, that act efficiently on xylan structures from woody plants, whereas molds use Bxls that efficiently degrade xylan from grass. These findings provide new insight into our understanding into the fungal efficient xylan degradation system.

## Experimental procedures

### Sequence analysis

Amino acid sequence of *Tr*Xyl3A was obtained from GenBank (CAA93248.1, http://www.ncbi.nlm.nih.gov) (40). Amino acid sequence of *Pc*Bxl3 was obtained from Joint Genome Institute (JGI) MycoCosm (Protein ID: 2919526) (41). The Blastp server (https://blast.ncbi.nlm.nih.gov/Blast.cgi?PAGE=Proteins) was used for calculation of identity. The amino acid sequences were aligned using Clustal W (42). Secondary structures were predicted by ESPript using the alignment and the native form of *Tr*Xyl3A (43).

### Protein preparations

*P. chrysosporium* strain K-3 was grown on Kremer and Wood medium (44) containing 2% cellulose (CF11; Whatman, Kent, UK) as the sole carbon source. Total RNA was extracted from approximately 100 mg of frozen mycelial powder using Isogen (Nippon Gene), and mRNA was purified from 1 μg of the total RNA using Oligotex-dT30 Super (Takara Bio), both according to the manufacturers’ instructions. Synthesis of first-strand cDNA from the mRNA was performed using a GeneRacer kit with SuperScript III reverse transcriptase (Invitrogen). The oligonucleotide primers, Pcxyn3_07481_3RACE (5′-GGTCGTCAGCCCAGATTTAAGCTAG-3′) and Pcxyn3_9257_5RACE (5′-GCGGGAACGAAGTAGCAGAGCTG-3′) for amplification of a cDNA fragment encoding *Pc*Bxl3, were designed based on the genomic sequences of *P. chrysosporium* available at Genome JGI. PCR was performed using KOD-Plus (TOYOBO). The cloned fragment was amplified with the gene-specific primers, Pcxyn3_9257_Met (5′- ATGGTCGCCAGCCCAGATTTAAGC-3′) and Pcxyn3_9257_Met (5′- GCTGCCTATGTACACTACCGTGGTTG -3′), and ligated into pPICZα A plasmid vector (Invitrogen) using EcoRI and NotI restriction recognition sites. After transformation of *Pichia pastoris* strain KM71H by electroporation, transformants were selected with Zeocin according to previous research (45, 46, 47).

pPICZα A plasmid vector encoding *Trxyl*3A between XhoI and NotI restriction recognition sites was optimized for *P. pastoris* and synthesized by Genscript. Subsequent procedures were as described above.

The recombinant proteins were produced using a 5 L jar fermenter (TSC-M5L; Takasugi Seisakusho) with methanol feed according to previous reports (45, 46, 47). The concentration of crude protein was quantified using Bradford Protein Assay (Bio-Rad) (48) using bovine serum albumin as standard. The yields of *Pc*Bxl3 and *Tr*Xyl3A were about 450 mg/L and 2500 mg/L, respectively. After 120 h, the medium was collected by centrifugation (30 min at 5000*g*), subjected to 100 kDa ultrafiltration, and concentrated using a 5 kDa filter (Merck Millipore).

The resulting solutions were purified by two-step column chromatography. For the characterization of *Pc*Bxl3 and *Tr*Xyl3A, Phenyl Toyopearl 650M (c.v. = 20.5 ml, Tosoh) was used for the first chromatography. After equilibration with 50 mM sodium acetate buffer (pH 5.0) containing 1 M ammonium sulfate (Wako), the enzymes were eluted with 50 mM sodium acetate buffer (pH 5.0). TSKgel DEAE-5PW (c.v. = 3.3 ml, Tosoh) was used for further purification. After equilibration with 50 mM Tris-HCl buffer (pH 8.0), the enzymes were eluted with 50 mM Tris-HCl buffer (pH 8.0) containing 1 M NaCl (Wako). The concentration of the purified proteins was quantified by NanoDrop2000 (Thermo Fisher Scientific)

For crystallization of *Pc*Bxl3, deglycosylation was conducted by endoglycosidase H from *Streptomyces plicatus* before the DEAE column chromatography. The pET 28 vector with the gene encoding endoglycosidase H was a generous gift from Professor Satoshi Kaneko. Transformation of *Escherichia coli* BL21(DE3) (New England Biolabs) was conducted according to the manufacturer’s instructions. After cultivation in LB medium, protein expression was induced with 100 μM IPTG at 37 °C. The medium containing cells was centrifuged at 10,000*g* for 10 min, and cell lysis was performed using BugBuster (Merck) according to the manufacturer’s instructions. The recombinant endoglycosidase H was purified using HisTrap HP column (GE). After equilibration with 20 mM Tris-HCl buffer (pH 7.0) containing 500 mM imidazole (Wako), the enzyme was eluted with 20 mM Tris-HCl buffer (pH 7.0). The protein solution of *Pc*Bxl3, without denaturing, was mixed with 0.2 mg purified endoglycosidase H per 1 mg of *Pc*Bxl3 and sodium acetate buffer (pH 6.0). The mixed solution was incubated at 37 °C for 24 h.

The protein used for crystallization of *Tr*Xyl3A was a kind gift from DuPont Industrial Bioscience. The protein was stored prior to crystallization experiments at 4 °C in a stock solution containing 149 mg/ml protein, 13% sorbitol, and 0.125% sodium benzoate in 0.1 M Sodium acetate (pH 5.0).

### Crystallization of Bxls

The crystallization of *Pc*Bxl3 was conducted by sitting-drop vapor diffusion method using a 96-well sitting drop plate (Greiner). For crystallization, 0.5 μl of 10 mg/ml *Pc*Bxl3 was mixed with 0.5 μl of the reservoir solution containing 0.2 M lithium sulfate, 0.1 M Tris (pH 8.5) and 40 v/v % PEG 400 (No.43 of JCSG + Suite, Qiagen). The plate was incubated at 293 K for 3 weeks. The reservoir solution for soaking contained 525 mM malic acid (pH 7.0, QIAGEN) and 20 v/v % PEG 3350 (Sigma-Aldrich). The mixture was incubated at 293 K for 1 week. The crystal was soaked in 40 v/v % PEG 400 (Sigma-Aldrich) and 100 mM X_2_ (Megazyme) for a short time.

The *Tr*Xyl3A protein stock solution was diluted to 10 mg/ml by adding 0.1 M sodium acetate buffer (pH 4.5) just prior to crystallization. Optimized *Tr*Xyl3A crystals for data collection were obtained by the hanging drop vapor diffusion method. Crystals for ligand-bound *Tr*Xyl3A data collection were obtained using the PACT screen (Qiagen) condition C4 (0.1 M PCB (pH 7.0) and 25% PEG 1500). 4-thioxyloboise was chemically synthesized according to the previous research (49). Soaking of 4-thioxylobiose to the crystals was done by 1 h incubation of *Tr*Xyl3A crystals in 0.095 M PCB (pH 7.0) and 33% PEG 1500, with 14 mM 4-thioxylobiose. Prior to data collection, the *Tr*Xyl3A crystals were briefly incubated in a cryoprotectant solution containing 30% PEG 3350 and 10% glycerol and then flash-frozen in liquid nitrogen.

### Data collection and structure determination

The dataset of a *Tr*Xyl3A crystal soaked with 4-thioxylobiose (to 2.1 Å resolution) was collected at beamline I911-5, at MAXII-lab, Sweden. All *Tr*Xyl3A diffraction data were processed using the data integration program Mosflm (50) and scaled using Scala in the CCP4i Software suite (51). The *Tr*Xyl3A 4-thioxylobiose ligand structure was solved by molecular replacement (MR) using the program Phaser (52, 53) using the nonligated *Tr*Xyl3A structure (PDB ID: 5A7M, chain B) as search model.

Structure refinement of the *Tr*Xyl3A ligand-bound structure was performed using the program REFMAC5 (54) and 5% of the data was excluded from the refinement for cross-validation and *R*_free_ calculations (55). Throughout the refinement, 2m*F*_o_-D*F*_c_ and m*F*_o_-D*F*_c_ sigma A weighted maps (56) were generated and inspected so that the model could be manually built and adjusted in Coot (57). The statistics of structure refinement is shown in Table 1.

Diffraction experiments for *Pc*Bxl3 crystals were conducted at the beamline of the Photon Factory (PF), High Energy Accelerator Research Organization. Diffraction data were collected on a DECTRIS PILATUS3 S 6M (Dectris). Crystals were cryocooled in a nitrogen gas stream to 95 K. The crystal data of free *Pc*Bxl3 were integrated and scaled using XDS installed in PREMO. The data for the crystal soaked in X_2_ solution were integrated and scaled using XDS and Aimless in the CCP4i2 program suite. Structural determination of *Pc*Bxl3 was conducted by molecular replacement with MOLREP in the CCP4i2 program suite. *N*-Acetyl-d-glucosamine, xylose, water molecules, and crystallization agents were modeled based on the electron density map and the coordination distances. The orientation of xylose molecule was determined by mimicking that of 5A7M at subsite −1. Refinement was conducted by Refmac5 in CCP4i2 (58) and Phenix.refine (59) in the Phenix program suite (version 1.18.2–3874, Lawrence Berkeley National Laboratory; 51).

The coordinates for the final *Tr*Xyl3A and *Pc*Bxl3 structure models, and the structure-factor amplitudes, have been deposited at the Protein Data Bank (PDB) (60, 61, 62), with access codes 5AE6 (*Tr*Xyl3A), and 7VC6 and 7VC7 (*Pc*Bxl3), respectively.

PyMOL (The PyMOL molecular Graphics System, version 2.2.3, Schrödinger, LLC) was used for structural drawings. Ligand–protein interaction was depicted using Ligplot+ (v.2.2) (63)

### Kinetics of Bxls

Xylobiose (X_2_), xylotriose (X_3_), xylotetraose (X_4_), and xylopentaose (X_5_) (Megazyme) were used as substrates. The reaction mixture consisted of enzyme, 5 mM sodium acetate buffer (pH 5.0), and various concentrations of xylooligosaccharides in the range from 50 μM to 5 mM. The concentrations of *Pc*Bxl3 and *Tr*Xyl3A were 30.2 nM and 26.6 nM, respectively. The reaction was performed at 30 °C for 30 min and stopped by heating the mixture at 95 °C for 5 min to prevent the reaction from continuing during quantification. Measurement of the amount of released xylose was performed by high-performance liquid chromatography (LC-2000 series; Jasco) with tandem columns of SUGAR-KS802 and 801 (Showa Denko). The samples were manually injected. The column oven was set at 70 °C. A Corona CAD detector (ESA Biosciences) was used.

### Subsite theory

Subsite theory is a simple theory describing the relationship between kinetics parameters and subsite affinities. The calculation for each subsite was conducted as described (23, 24, 25).

### Docking simulation

Simulation was conducted using the Autodock Vina program (1.1.2) (64) in Chimera (version1.14.0) (65). The model structures of X_2_ and X_3_ were built using the Sweet2 server (66, 67).

## Data availability

The structures presented in this paper have all been deposited in the Protein Data Bank (PDB) with the following codes: 5AE6 (crystal structure of *Tr*Xyl3A bound with thioxylobiose), 7VC6 (native form of *Pc*Bxl3), and 7VC7 (crystal structure of *Pc*Bxl3 bound with xylose).

## Supporting information

This article contains supporting information (42).

## Conflict of interest

The authors declare that there is no conflict of interests with the contents of this article.

## References

[1] Saha B.C. (2003). Hemicellulose bioconversion. J. Ind. Microbiol. Biotechnol..

[2] Hori C., Gaskell J., Igarashi K., Samejima M., Hibbett D., Henrissat B., Cullen D. (2013). Genomewide analysis of polysaccharides degrading enzymes in 11 white- and brown-rot Polyporales provides insight into mechanisms of wood decay. Mycologia.

[3] Biely P., Singh S., Puchart V. (2016). Towards enzymatic breakdown of complex plant xylan structures: State of the art. Biotechnol. Adv..

[4] Bromley J.R., Busse-Wicher M., Tryfona T., Mortimer J.C., Zhang Z., Brown D.M., Dupree P. (2013). GUX1 and GUX2 glucuronyltransferases decorate distinct domains of glucuronoxylan with different substitution patterns. Plant J..

[5] Lombard V., Golaconda Ramulu H., Drula E., Coutinho P.M., Henrissat B. (2014). The carbohydrate-active enzymes database (CAZy) in 2013. Nucleic Acids Res..

[6] Kawai R., Igarashi K., Kitaoka M., Ishii T., Samejima M. (2004). Kinetics of substrate transglycosylation by glycoside hydrolase family 3 glucan (1→3)-β-glucosidase from the white-rot fungus *Phanerochaete chrysosporium*. Carbohydr. Res..

[7] Yoshida E., Hidaka M., Fushinobu S., Koyanagi T., Minami H., Tamaki H., Kitaoka M., Katayama T., Kumagai H. (2010). Role of a PA14 domain in determining substrate specificity of a glycoside hydrolase family 3 β-glucosidase from *Kluyveromyces marxianus*. Biochem. J..

[8] Martinez D., Berka R.M., Henrissat B., Saloheimo M., Arvas M., Baker S.E., Chapman J., Chertkov O., Coutinho P.M., Cullen D., Danchin E.G.J., Grigoriev I.V., Harris P., Jackson M., Kubicek C.P. (2008). Genome sequencing and analysis of the biomass-degrading fungus *Trichoderma reesei* (syn. *Hypocrea jecorina*). Nat. Biotechnol..

[9] Tenkanen M., Luonteri E., Teleman A. (1996). Effect of side groups on the action of β-xylosidase from *Trichoderma reesei* against substituted xylo-oligosaccharides. FEBS Lett..

[10] Bischof R.H., Ramoni J., Seiboth B. (2016). Cellulases and beyond: The first 70 years of the enzyme producer *Trichoderma reesei*. Microb. Cell Fact..

[11] Gene A., An T., Lecula M.O., Tech B.I.O., Ogasawara N.W. (2006). Cloning, functional expression and promoter analysis of xylanase III gene from *Trichoderma reesei*. Appl. Microbiol. Biotechnol..

[12] Tenkanen M. (1992). Two major xylanases of *Trichoderma reesei*. Enzyme Microb. Technol..

[13] Biely P., Puchart V., Stringer M.A., Mørkeberg Krogh K.B.R. (2014). *Trichoderma reesei* XYN VI – a novel appendage-dependent eukaryotic glucuronoxylan hydrolase. FEBS J..

[14] Herrmann M.C., Vrsanska M., Jurickova M., Hirsch J., Biely P., Kubicek C.P. (1997). The β-d-xylosidase of *Trichoderma reesei* is a multifunctional β-d-xylan xylohydrolase. Biochem. J..

[15] Martinez D., Larrondo L.F., Putnam N., Sollewijn Gelpke M.D., Huang K., Chapman J., Helfenbein K.G., Ramaiya P., Detter J.C., Larimer F., Coutinho P.M., Henrissat B., Berka R., Cullen D., Rokhsar D. (2004). Genome sequence of the lignocellulose degrading fungus *Phanerochaete chrysosporium* strain RP78. Nat. Biotechnol..

[16] Sakuragi K., Hori C., Igarashi K., Samejima M. (2018). Secretome analysis of the basidiomycete *Phanerochaete chrysosporium* grown on ammonia-treated lignocellulosic biomass from birch wood. J. Wood Sci..

[17] Hrmova M., Varghese J.N., De Gori R., Smith B.J., Driguez H., Fincher G.B. (2001). Catalytic mechanisms and reaction intermediates along the hydrolytic pathway of a plant β-d-glucan glucohydrolase. Structure.

[18] Dan S., Marton I., Dekel M., Bravdo B.A., He S., Withers S.G., Shoseyov O. (2000). Cloning, expression, characterization, and nucleophile identification of family 3, *Aspergillus niger* β-glucosidase. J. Biol. Chem..

[19] Li Y.K., Chir J., Tanaka S., Chen F.Y. (2002). Identification of the general acid/base catalyst of a family 3 *β*-glucosidase from *Flavobacterium meningosepticum*. Biochemistry.

[20] Rose I.A., Hanson K.R., Wilkinson K.D., Wimmer M.J. (1980). A suggetion for naming faces of ring compounds. Proc. Natl. Acad. Sci. U. S. A..

[21] Wurman-Rodrich J. (2017).

[22] Karkehabadi S., Helmich K.E., Kaper T., Hansson H., Mikkelsen N.E., Gudmundsson M., Piens K., Fujdala M., Banerjee G., Scott-Craig J.S., Walton J.D., Phillips G.N., Sandgren M. (2014). Biochemical characterization and crystal structures of a fungal family 3 β-glucosidase, Cel3A from *Hypocrea jecorina*. J. Biol. Chem..

[23] Hiromi K. (1970). Interpretation of dependency of rate parameters on the degree of polymerization of substrate in enzyme-catalyzed reactions. Evaluation of subsite affinities of exo-enzyme. Biochem. Biophys. Res. Commun..

[24] Hiromi K., Nitta Y., Numata C., Ono S. (1973). Subsite affinities of glucoamylase: Examination of the validity of the subsite theory. Biochim. Biophys. Acta.

[25] Hiromi K., Ohnishi M., Tanaka A. (1983). Subsite structure and ligand binding mechanism of glucoamylase. Mol. Cell. Biochem..

[26] Kita A., Matsui H., Somoto A., Kimura A., Takata M., Chiba S. (1991). Substrate specificity and subsite affinities of crystalline α-glucosidase from *Aspergillus niger*. Agric. Biol. Chem..

[27] Bonnin E., Vigouroux J., Thibault J.F. (1997). Kinetic parameters of hydrolysis and transglycosylation catalyzed by an exo-β-(1,4)-galactanase. Enzyme Microb. Technol..

[28] Opassiri R., Hua Y., Wara-aswapati O., Akiyama T., Svasti J. (2004). β-glucosidase, exo-β-glucanase and pyridoxine transglucosylase activities of rice Bglu1. Biochem. J..

[29] Kulminskaya A.A., Thomsen K.K., Shabalin K.A., Sidorenko I.A., Eneyskaya E.V., Savel’Ev A.N., Neustroev K.N. (2001). Isolation, enzymatic properties, and mode of action of an exo-1,3-β-glucanase from *Trichoderma viride*. Eur. J. Biochem..

[30] Matsuzawa T., Watanabe M., Nakamichi Y., Fujimoto Z., Yaoi K. (2019). Crystal structure and substrate recognition mechanism of *Aspergillus oryzae* isoprimeverose-producing enzyme. J. Struct. Biol..

[31] Nakajima M., Yoshida R., Miyanaga A., Abe K., Takahashi Y., Sugimoto N., Toyoizumi H., Nakai H., Kitaoka M., Taguchi H. (2016). Functional and structural analysis of a β-glucosidase involved in β-1,2-glucan metabolism in *Listeria innocua*. PLoS One.

[32] Ishiguro R., Tanaka N., Abe K., Nakajima M., Maeda T., Miyanaga A., Takahashi Y., Sugimoto N., Nakai H., Taguchi H. (2017). Function and structure relationships of a β-1,2-glucooligosaccharide-degrading β-glucosidase. FEBS Lett..

[33] Floudas D., Binder M., Riley R., Barry K., Blanchette R.T., Henrissat B., Martínez A.T., Otillar R., Spatafora J.W., Yadav J.S., Aerts A., Benoit I., Boyd A., Carlson A., Copeland A. (2012). The paleozoic origin of enzymatic lignin decomposition reconstructed from 31 fungal genomes. Science.

[34] Schröder S.P., De Boer C., McGregor N.G.S., Rowland R.J., Moroz O., Blagova E., Reijngoud J., Arentshorst M., Osborn D., Morant M.D., Abbate E., Stringer M.A., Krogh K.B.R.M., Raich L., Rovira C. (2019). Dynamic and functional profiling of xylan-degrading enzymes in *Aspergillus* secretomes using activity-based probes. ACS Cent. Sci..

[35] Carafa A., Duckett J.G., Nox J.P., Ligrone R. (2005). Distribution of cell-wall xylans in bryophytes and tracheophytes: New insights into basal interrelationships of land plants. New Phytol..

[36] Martínez-Abad A., Berglund J., Toriz G., Gatenholm P., Henriksson G., Lindström M., Wohlert J., Vilaplana F. (2017). Regular motifs in xylan modulate molecular flexibility and interactions with cellulose surfaces. Plant Physiol..

[37] Biely P., Cziszárová M., Uhliariková I., Agger J.W., Li X.L., Eijsink V.G.H., Westereng B. (2013). Mode of action of acetylxylan esterases on acetyl glucuronoxylan and acetylated oligosaccharides generated by a GH10 endoxylanase. Biochim. Biophys. Acta.

[38] Biely P., Puls J., Schneider H. (1985). Acetyl xylan esterases in fungal cellulolytic systems. FEBS Lett..

[39] Busse-Wicher M., Li A., Silveira R.L., Pereira C.S., Tryfona T., Gomes T.C.F., Skaf M.S., Dupree P. (2016). Evolution of xylan substitution patterns in gymnosperms and angiosperms: Implications for xylan interaction with cellulose. Plant Physiol..

[40] Benson D.A., Cavanaugh M., Clark K., Karsch-Mizrachi I., Lipman D.J., Ostell J., Sayers E.W. (2013). GenBank. Nucleic Acids Res..

[41] Grigoriev I.V., Nikitin R., Haridas S., Kuo A., Ohm Robin., Otillar R., Riley R., Salamov A., Zhao X., Korzeniiewski F., Smirnova T., Nordberg H., Dubchak I., Shabalov I. (2013). MycoCosm portal: Gearing up for 1000 fungal genomes. Nucleic Acids Res..

[42] Larkin M.A., Blackshields G., Brown N.P., Chenna R., Mcgettigan P.A., McWilliam H., Valentin F., Wallace I.M., Wilm A., Lopez R., Thompson J.D., Gibson T.J., Higgins D.G. (2007). Clustal W and clustal X version 2.0. Bioinformatics.

[43] Robert X., Gouet P. (2014). Deciphering key features in protein structures with the new ENDscript server. Nucleic Acids Res..

[44] Kremer S.,M., Wood P.,M. (1992). Evidence that cellobiose oxidase from *Phanerochaete chrysosporium* is primarily an fn(III) reductase: Kinetic comparison with neutrophil NADPH oxidase and yeast flavocytichrome *b*_2_. Eur. J. Biochem..

[45] Igarashi K., Ishida T., Hori C., Samejima A. (2008). Characterization of an endoglucanase belonging to a new subfamily of glycoside hydrolase family 45 of the basidiomycete *Phanerochaete chrysosporium*. Appl. Environ. Microbiol..

[46] Igarashi K., Marauyama M., Nakamura A., Ishida T., Wada M., Samejima A. (2012). Degradation of crystalline celluloses by *Phanerochaete chrysosporium* cellobiohydrolase II (Cel6A) heterologously expressed in methylotrophic yeast *Pichia pastoris*. J. Appl. Glycosci..

[47] Matsuyama K., Sunagawa N., Igaarashi K. (2020). Mutation of cystein residues increases heterologous expression of peach expansin in the methylotriphic yeast *Pichia pastoris*. Plant Biotechnol..

[48] Bradford M.M. (1976). A rabid and sentive method for the quantification of microgram quantities of protein utilizing the principle of protein-dye binding. Anal. Biochem..

[49] Rho D., Desrochers M., Jurasek L., Drigeuz H., Defaye J. (1982). Induction of cellulase in *Schizophyllum commune*: Thiocellobiose as a new inducer. J. Bacteriol..

[50] Leslie A. (2006). The integration of macromolecular diffraction data. Acta Crystallogr. D Biol. Crystallogr..

[51] Collaborative Computational Project, Number 4 (1994). The *CCP*4 suite: Programs for protein crystallography. The integration of macromolecular diffraction data. Acta Crystallogr. D Biol. Crystallogr..

[52] McCoy A.J. (2007). Solving structures of protein complexes by molecular replacement with *Phaser*. Acta Crystallogr. D Biol. Crystallogr..

[53] McCoy A.J., Grosse-Kunstleve R.W., Adams P.D., Winn M.D., Storoni L.C., Read R.J. (2007). *Phaser* crystallographic software. J. Appl. Cryst..

[54] Murshudov G.N., Vagin A.A., Dodson E.J. (2007). Refinement of macromolecular structures by the maximum-likelihood method. Acta Crystallogr. D Biol. Crystallogr..

[55] Brünger A.T. (1992). Free *R* value: A novel statiscal quantity for assessing the accuracy of crystal structures. Nature.

[56] Pannu N.S., Read R.J. (1996). Improved structure refinement through maximum likelihood. Acta Cryst..

[57] Emsley P., Cowtan K. (2004). *Coot*: Model-building tools for molecular graphics. Acta Crystallogr. D Biol. Crystallogr..

[58] Winn M.D., Ballard C.C., Cowtan K.D., Dodson E.J., Emsley P., Evans P.R., Keegan R.M., Krissinel E.B., Leslie A.G.W., McCoy A., McNicholas S.J., Murshudov G.N., Pannu N.S., Potterton E.A., Powell H.R. (2011). Overview of the CCP4 suite and current developments. Acta Crystallogr. D Biol. Crystallogr..

[59] Afonine P.V., Grosse-Kunstleve R.W., Echols N., Headd J.J., Moriarty N.W., Mustyakimov M., Terwilliger T.C., Urzhumtsev A., Zwart P.H., Adams P.D. (2012). Towards automated crystallographic structure refinement with phenix.refine. Acta Crystallogr. D Biol. Crystallogr..

[60] Bernstein F.C., Koetzle T.,.F., Williams G.J.B., Meyer E.F., Brice M.D., Rodgers J.R., Kennard O., Shimanouchi T., Tasumi M. (1977). The Protein Data Bank: A computer-based achival file for macromolecular structures. J. Mol. Biol..

[61] Sussman J.L., Lin D., Jiang J., Manning N.O., Prilusky J., Ritter O., Abola E.E. (1998). Protein Data Bank (PDB): Database of three-dimensional structural information of biological macromolecules. Acta Crystallogr. D Biol. Crystallogr..

[62] Keller P.A., Henrick K., McNeil P., Moodle S., Barton G.J. (1998). Deposition of macromolecular structures. Acta Cryst..

[63] Laskowski R.A., Swindells M.B. (2011). LigPlot+: Multiple ligand-protein interaction diagrams for drug discovery. J. Chem. Inf. Model..

[64] Oleg T., Arthur J.O. (2010). AutoDock Vina: Improving the speed and accuracy of docking with a new scoring function, efficient optimization and multithreading. J. Comput. Chem..

[65] Pettersen E.F., Goddard T.D., Huang C.C., Couch G.S., Greenblatt D.M., Meng E.C., Ferrin T.E. (2004). UCSF Chimera–a visualization system for exploratory research and analysis. J. Comput. Chem..

[66] Bohne A., Lang E., von Der Lieth C.W. (1998). W3-SWEET: Carbohydrate modeling by internet. J. Mol. Model..

[67] Bohne A., Lang E., von Der Lieth C.W. (1999). SWEET-WWW-based rapid 3D construction of oligo- and polysaccharides. Bioinformatics.

