# Supplementary material for: Comparison of glycoside hydrolase family 3 β-xylosidases from basidiomycetes and ascomycetes reveals evolutionarily distinct xylan degradation systems
Source: J Biol Chem. 2022 Feb 1;298(3):101670. doi: 10.1016/j.jbc.2022.101670 (PMC8913315; doi:10.1016/j.jbc.2022.101670)
Supplement: Supplemental Figure S1 [file mmc1.pdf]

Supplementary Materials for

Comparison of Glycoside Hydrolase family 3 β-xylosidases from basidiomycetes and ascomycetes reveals evolutionarily distinct xylan degradation systems

Keisuke Kojima[1], Naoki Sunagawa[1], Nils Egil Mikkelsen[2], Henrik Hansson[2], Saeid Karkehabadi[2], Masahiro Samejima[1,3], Mats Sandgren[2] and Kiyohiko Igarashi[1,4*]

Figure legend

Figure S1. Sequence alignment of *Tr*Xyl3A and *Pc*Bxl3

The amino acid sequences of *Tr*Xyl3A and *Pc*Bxl3 were aligned by Clustal W (42). The upper sequence is *Tr*Xyl3A and the lower is *Pc*Bxl3.

```
PcBxl3     1 ---------------------------AFPDCANGPLKSNLVCNASADPVSRAKALVDAL
TrXyl3A    1 QNNQTYANYSAQGQPDLYPETLATLTLSFPDCEHGPLKNNLVCDSSAGYVERAQALISLF

PcBxl3    34 TLEELVNNTVNASPGVPRVGLPPYNWWSEALHGVARSPGTNFSTVPGSPFSSATSFPQPI
TrXyl3A   61 TLEELILNTQNSGPGVPRLGLPNYQVWNEALHGLDRAN----FATKGGQFEWATSFPMPI

PcBxl3    94 ILGATFDDDLIHSIATVISTEARAFNNAGRAGLDFFTPNINPFKDPRWGRGQETPGEDPY
TrXyl3A  117 LTTAALNRTLIHQIADIISTQARAFSNSGRYGLDVYAPNVNGFRSPLWGRGQETPGEDAF

PcBxl3   154 HIA-QYVYQLITGLQGGLSPDPYYKVVADCKHFAGYDLEDWHGNNRMAFNAVISTQDLAE
TrXyl3A  177 FLSSAYTYEYITGIQGGVDP-EHLKVAATVKHFAGYDLENWNNQSRLGFDAIITQQDLSE

PcBxl3   213 FYTPSFQSCVRDAHVGSVMCSYNAVNGVPSCASPYLLQDLIRDHFGLGDG-WITSDCDAV
TrXyl3A  236 YYTPQFLAAARYAKSRSLMCAYNSVNGVPSCANSFFLQTLLRESWGFPEWGYVSSDCDAV

PcBxl3   272 DNVFDPHNYTSTLVNASAVSLKAGTDVDCGTTYSQTLVDAVNQKLVTEDDVKTSMVRLYS
TrXyl3A  296 YNVFNPHDYASNQSSAAASSLRAGTDIDCGQTYPWHLNESFVAGEVSRGEIERSVTRLYA

PcBxl3   332 SLVRLGYFDSPENQPWRQLGWADVNTPSAQALALTAAEEGVVLLKNDGTLPLSRRIKHIA
TrXyl3A  356 NLVRLGYFD--KKNQYRSLGWKDVVKTDAWNISYEAAVEGIVLLKNDGTLPLSKKVRSIA

PcBxl3   392 VVGPWANATTQMQGNYQGIAPFLISPLQALQDAGFHVSFANGTAINSTDTSGFASALMAA
TrXyl3A  414 LIGPWANATTQMQGNYYGPAPYLISPLEAAKKAGYHVNFELGTEIAGNSTTGFAKAIAAA

PcBxl3   452 KAADAIVFAGGIDETIESEGHDRDSIEWPGNQLDLIEQLAALRKPLIVLQMGGGQVDSSS
TrXyl3A  474 KKSDAIIYLGGIDNTIEQEGADRTDIAWPGNQLDLIKQLSEVGKPLVVLQMGGGQVDSSS

PcBxl3   512 LKASKAVNALLWGGYPGQSGGTAIVNILTGKTAPSGRLPITQYPAAYVDAIPMTDMALRP
TrXyl3A  534 LKSNKKVNSLVWGGYPGQSGGVALFDILSGKRAPAGRLVTTQYPAEYVHQFPQNDMNLRP

PcBxl3   572 SS-SSPGRTYKWYTGTPVFDFGFGLHYTSFKLSWAASPPSRFDISSLVAGAKHAGVAFTD
TrXyl3A  594 DGKSNPGQTYIWYTGKPVYEFGSGLFYTTFKETLASHPKSLKFNTSSILSAPHPGYTYSE

PcBxl3   631 LAPLFTFHVAVKNSGKVTSDYVALLFAHTT-VGPSPAPQQELVAYTRVKGITPGRTATAA
TrXyl3A  654 QIPVFTFEANIKNSGKTESPYTAMLFVRTSNAGPAPYPNKWLVGFDRLADIKPGHSSKLS

PcBxl3   690 LSVTLGSIARVDESGVRSLYPGKYSVWVDTTREIMHTFELTGKTTQILGWPQPR------
TrXyl3A  714 IPIPVSALARVDSHGNRIVYPGKYELALNTDESVKLEFELVGEEVTIENWPLEEQQIKDA

PcBxl3       ----
TrXyl3A  774 TPDA
```

Figure S1
